# Nonstoichiometric oxygen in Mn–Ga–O spinels: reduction features of the oxides and their catalytic activity

**DOI:** 10.1039/c7ra11557a

**Published:** 2018-03-27

**Authors:** O. A. Bulavchenko, O. S. Venediktova, T. N. Afonasenko, P. G. Tsyrul'nikov, A. A. Saraev, V. V. Kaichev, S. V. Tsybulya

**Affiliations:** Boreskov Institute of Catalysis SB RAS Lavrentieva Ave. 5 Novosibirsk 630090 Russia isizy@catalysis.ru; Novosibirsk State University Pirogova Str. 2 Novosibirsk 630090 Russia; Institute of Hydrocarbons Processing SB RAS Neftezavodskaya Str. 54 Omsk 644040 Russia

## Abstract

The subject of this study was the content of oxygen in mixed oxides with the spinel structure Mn_1.7_Ga_1.3_O_4_ that were synthesized by coprecipitation and thermal treatment in argon at 600–1200 °C. The study revealed the presence of excess oxygen in “low-temperature” oxides synthesized at 600–800 °C. The occurrence of superstoichiometric oxygen in the structure of Mn_1.7_Ga_1.3_O_4+*δ*_ oxide indicates the formation of cationic vacancies, which shows up as a decreased lattice parameter in comparison with “high-temperature” oxides synthesized at 1000–1200 °C; the additional negative charge is compensated by an increased content of Mn^3+^ cations according to XPS. The low-temperature oxides containing excess oxygen show a higher catalytic activity in CO oxidation as compared to the high-temperature oxides, the reaction temperature was 275 °C. For oxides prepared at 600 and 800 °C, catalytic activity was 0.0278 and 0.0048 cm^3^ (CO) per g per s, and further increase in synthesis temperature leads to a drop in activity to zero. The process of oxygen loss by Mn_1.7_Ga_1.3_O_4+*δ*_ was studied in detail by TPR, *in situ* XRD and XPS. It was found that the hydrogen reduction of Mn_1.7_Ga_1.3_O_4+*δ*_ proceeds in two steps. In the first step, excess oxygen is removed, Mn_1.7_Ga_1.3_O_4+*δ*_ → Mn_1.7_Ga_1.3_O_4_. In the second step, Mn^3+^ cations are reduced to Mn^2+^ in the spinel structure with a release of manganese oxide as a single crystal phase, Mn_1.7_Ga_1.3_O_4_ → Mn_2_Ga_1_O_4_ + MnO.

## Abstract 2

The hydrogen reduction of Mn_1.7_Ga_1.3_O_4+*δ*_ proceeds in two steps. In the first step, excess oxygen is removed, Mn_1.7_Ga_1.3_O_4+*δ*_ → Mn_1.7_Ga_1.3_O_4_. In the second step, Mn^3+^ cations are reduced to Mn^2+^ in the spinel structure and formation of MnO, Mn_1.7_Ga_1.3_O_4_ → Mn_2_Ga_1_O_4_ + MnO.

## Graphical abstract

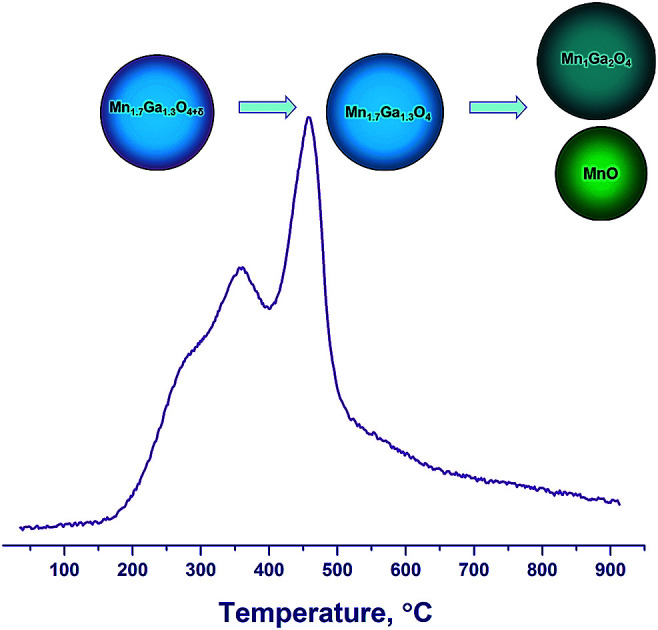

## Introduction

The ability of manganese cations to change their oxidation state makes Mn-containing oxides efficient catalysts for various processes: oxidation of hydrocarbons, CO, and VOCs^1–6^ and the selective reduction of NO_*x*_ with NH_3_.^7^

Of special interest are Mn-containing oxides with a spinel structure that possess a high thermal stability and are able to intercalate additional cations. The catalytic properties of Mn-containing oxides were examined in different processes: Mn–Co oxides are promising for the oxidation of CO, propene,^8^ and benzyl alcohol,^9^ as materials for fuel cells and oxygen reduction/evolution electrocatalysts;^10,11^ Ga–Co–Mn can be employed for visible-light-driven water oxidation;^12^ Mn–Al spinels are precursors of the active component of catalysts for the oxidation of CO and hydrocarbons;^13^ Zn–Mn–Al oxides are used for the reduction of nitrobenzene to nitroazobenzene;^14^ and Mn–Fe as Fenton catalysts toward catalytic degradation of highly concentrated methylene blue.^15^

The spinel structure is described by a general formula AB_2_O_4_, where A is a divalent cation, and B is a trivalent one. In a normal spinel, divalent cations A are located in tetrahedral positions, and trivalent cations B located in octahedral positions; however, in some compounds a partial or complete inversion may take place: divalent cation A resides in the octahedral position, while trivalent B in the tetrahedral one. Nonstoichiometry of the metal/oxygen ratio is commonly implemented in the spinel structure *via* the cationic vacancies with preservation of the closest oxygen packing, A_1−*x*1_B_2−*x*2_[]_*y*_O_4_; in this case, the chemical formula can be written as AB_2_O_4+*δ*_, hence it follows that the structure includes superstoichiometric (excess) oxygen, which under certain conditions can pass to the gas phase and become reactive.

A relation between oxygen nonstoichiometry in Mn-containing oxides with the spinel structure and their catalytic properties was investigated only in several works. In particular it was shown that the Mn^3+^ cation in Zn_1−*x*_Mn_*x*_Al_2_O_4+*δ*_ (0 < *x* < 1) is the active site for reduction of nitrobenzene,^16^ which may occur only in the case of partial inversion of spinel and appearance of cationic vacancies.^14^ For Mn–Co oxides, the activity in the oxidation of benzyl alcohol depends on the concentration of trivalent cations and, accordingly, on the oxygen content in the exposed planes and in the sub-layer.^9^

In this connection, it seems important to examine the effect of oxygen nonstoichiometry on the catalytic properties in oxidation reactions. On Mn-containing oxides, oxidation reactions are known to proceed through the Mars–van Krevelen mechanism,^9^ according to which in the first step lattice oxygen oxidizes the substrate which is accompanied by generation of an oxygen vacancy, and in the second step the reduced catalyst is reoxidized by gas-phase molecular oxygen. To investigate the effect of oxygen nonstoichiometry in Mn-containing oxides on the catalytic properties, we placed the manganese cation in the redox inert matrix of gallium oxide, which is able to form spinel structures. The oxidation of CO served as a model reaction. Previously, we investigated the conditions of formation of Mn–Ga oxides in a wide range of Mn/Ga cation ratio from 2 to 0.5 and calcination temperatures 600–1200 °C.^17^ This paper considers in detail the redox properties of Mn–Ga oxides with the spinel structure and the reduction of Mn cations in spinel by *in situ* XRD, XPS, and TPR, as well as the effect of excess oxygen on the activity in the oxidation of CO.

## Experimental

### Preparation

The calculated amount of Ga(NO_3_)_3_ and Mn(NO_3_)_2_ aqueous solutions was poured into a round-bottom flask. Precipitation was carried out under stirring with a gradual addition of a 5 M NH_4_OH solution to bring the pH of the solution to 9. A mechanical stirrer rotated at 450 rpm. After a subsequent aging at 60 °C for 2 h, the precipitate was filtered, washed with distilled water on a filter to pH 6, and dried at 120 °C for 2 h.

The samples with Mn : Ga = 1.7 : 1.3 were calcined in an inert argon medium at 600, 800, 1000, and 1200 °C. Calcination was performed with a gas flow rate of 50–60 mL min^−1^ for 4 h. This was followed by cooling in the inert medium at a rate of 10 mL min^−1^ until reaching the room temperature. A series of samples with Mn : Ga = 1.7 : 1.3 was chosen for further studies as the X-ray nearly single-phase for all temperatures.^17^

Simple oxides Mn_2_O_3_ and Mn_3_O_4_ were synthesized from Mn(NO_3_)_2_ by calcination at 650 and 1200 °C in air for 4 h.

### XRD

Powder X-ray diffraction was carried out using a Bruker D8 Advance Diffractometer (Germany) with the CuK_α_ radiation (*λ* = 1.5418 Å) in the *θ*/2*θ* geometry. The diffractometer was equipped with a LynxEye linear semiconductor detector. A nickel filter was used to eliminate the CuK_β_ component. A 2*θ* range from 15 to 80° was scanned using a step of 0.05° and a counting time of 2 s at each point.

The diffraction data were interpreted using software programs and databases. In particular, the phase composition was determined with the use of PDF-4+ powder diffraction database.^18^ Sizes of coherent scattering region (CSR) were calculated by the Scherrer formula using 311 reflection of spinel.^19^

### *In situ* XRD

*In situ* X-ray diffraction study was carried out under hydrogen reduction conditions on a D8 Advance diffractometer equipped with a reaction chamber XRK-900 (Anton Paar, Austria). The hydrogen flow rate was 40 mL min^−1^; and heating rate, 10° min^−1^.

### XPS

X-ray photoelectron spectroscopy was applied for the chemical analysis of the catalysts after reduction in hydrogen. XPS studies were performed on an X-ray photoelectron spectrometer (SPECS Surface Nano Analysis GmbH, Germany) equipped with a XR-50M X-ray source with a twin Al/Ag anode, a FOCUS-500 X-ray monochromator, a PHOIBOS-150 hemispherical electron energy analyzer, and a high-pressure cell. The core-level spectra were obtained using the monochromatic AlKα radiation (*hν* = 1486.74 eV) under ultrahigh vacuum conditions. Charge correction was performed by setting the Ga 2p_3/2_ peak at 1117.9 eV. Relative element concentrations were determined from the integral intensities of XPS peaks using the cross-sections according to Scofield. For a detailed analysis, the spectra were fitted into several peaks after the background subtraction by the Shirley method. The fitting procedure was performed using the CasaXPS software.^20^ The line shapes were approximated by the multiplication of Gaussian and Lorentzian functions.

The Mn–Ga sample synthesized at 600 °C was treated in the high-pressure cell by the following procedure. Loading of the sample, evacuation to ultrahigh vacuum (10^−7^ mbar), and admission of a gas (hydrogen, oxygen or argon, ∼1000 Torr, cell volume ∼ 0.2 L); heating to a specified temperature for 10 min and treatment at this temperature for 30 min; evacuation of the cell and cooling to room temperature in a vacuum; recording of XP spectra.

### Catalytic tests

Catalytic tests were performed in a flow regime in a glass reactor 170 mm in length and 10 mm in diameter. The initial gas mixture composition was 1% CO, 2% O_2_, 8% N_2_, and He the rest. For changing the CO conversion, the flow rate of the gas mixture was varied in the range of 200–570 mL min^−1^. The oxidation of CO was carried out at 275 °C. The reactant mixture before and after the reactor was analyzed by a chromatograph equipped with a packed column (zeolite CaA, 3 m) and a thermal conductivity detector.

Catalytic activity was calculated from the CO conversion at different flow rates, taking into account the mass of the catalyst, according to the formula:*W*(CO) = [*C*_0_ − *C*_cur_] × *V*/(60 × *m*_cat_), [cm^3^ (CO) per g per s]*C*_cur_ = *C*_0_ × (1 − (*P*_0_ − *P*_cur_/*P*_0_)),where *P*_0_ is the peak area corresponding to the initial concentration of CO in the reactant mixture; *P*_cur_ is the peak area corresponding to the current concentration of CO at the reactor outlet; *C*_cur_ is the current concentration of CO in the mixture, vol%; *C*_0_ is the initial concentration of CO in the mixture (*C*_0_ = 1 vol%); *V* is the feed rate of the reactant mixture, mL min^−1^; and *m*_cat_ is the mass of a catalyst, g.

To compare the samples, their activities must be extrapolated to the same conversion of CO (in our case, 50% conversion):*W*(CO)_50%_ = 0.5 × *W*/*C*^av^_cur_,where *C*^av^_cur_ = (*C*_0_ + *C*_cur_)/2, (%)

Before the catalytic tests, the samples were mixed with a γ-Al_2_O_3_ binder (inactive under the used conditions) in a weight ratio of 1 : 1, pelletized and fractionated. The tests were performed with the fraction 0.8–1.4 mm. Effect of mixing with Al_2_O_3_ was taken into account during the calculations of catalytic activity.

### TPR

The temperature-programmed reduction in hydrogen (TPR-H_2_) was performed with 100 mg of a sample in a quartz reactor using a flow setup equipped with a thermal conductivity detector. The reducing mixture (10 vol% of H_2_ in Ar) was fed at 40 mL min^−1^. The rate of heating from room temperature to 900 °C was approximately 10 °C min^−1^.

### TG

The experiments were made on a STA 409 PC Luxx (Netzsch) derivatograph. Concentrations of the gas mixture components were measured using a QMS-200 mass spectrometer. Samples were heated to 1000 °C at a rate of 10 °C min^−1^. Argon flow rate was 70 mL min^−1^.

## Results and discussion

### State of the initial samples

#### XRD and TG in argon

Phase composition of samples with Mn : Ga ratio 1.7 : 1.3 synthesized at 600–1200 °C in argon corresponds mainly to the Mn_3−*x*_Ga_*x*_O_4_ spinel phase [JCPDS no. 38-0181]. Lattice parameters and average CSR sizes are listed in Table 1. One can see that CSR and lattice parameters of spinel increase with temperature. The origin of differences in the lattice parameters was discussed in details in our previous work^17^ and is related to the presence of excess oxygen.

**Table 1:** Characteristics of the samples: lattice parameter, average CSR size, weight loss, and excess oxygen *δ* in Mn_1.7_Ga_1.3_O_4+*δ*_ estimated by TG in argon

Calcination temperature of oxide, °C	Lattice parameter of Mn_1.7_Ga_1.3_O_4_, Å	CSR, Å	Weight loss, %	Estimated oxygen excess *δ* in Mn_1.7_Ga_1.3_O_4+*δ*_
600	8.413(4)	100	1.65	0.26
800	8.462(1)	350	0.5	0.08
1000	8.481(1)	650	0	0.00
1200	8.478(1)	>1000	0	0.00

Table 1 lists weight losses of Mn_1.7_Ga_1.3_O_4_ 600–1200 °C samples upon heating in an argon flow from 25 to 1000 °C. As the calcination temperature is raised, weight losses decrease from 1.65% for the oxide synthesized at 600 °C to 0% for 1200 °C. The oxygen content in spinel was estimated under the assumption that weight losses are related to the loss of excess oxygen in comparison with the stoichiometric ratio M : O = 3 : 4, where M is Ga or Mn. Such oxygen is weakly bound and can readily pass into the gas phase when the temperature is increased and/or the oxygen partial pressure is decreased. According to these data, the content of excess oxygen decreases with increasing the treatment temperature.

According to XRD date upon heating to 1000 °C in argon, the spinel structure is retained, but the lattice parameter increases from 8.413(4) to 8.460(1) Å, which can be attributed to the observed oxygen losses.^17^

#### XPS

The surface state of the tested mixed oxides was characterized by XPS. The binding energy scale for the MnGa-600 sample was calibrated against the C 1s peak of carbon (*E*_b_ = 284.8 eV); therewith, the binding energy of the Ga 2p_3/2_ peak was 1117.9 eV. For all other samples, the binding energy scale was calibrated against the internal standard using the Ga 2p_3/2_ peak (*E*_b_ = 1117.9 eV).

**Fig. 1:**
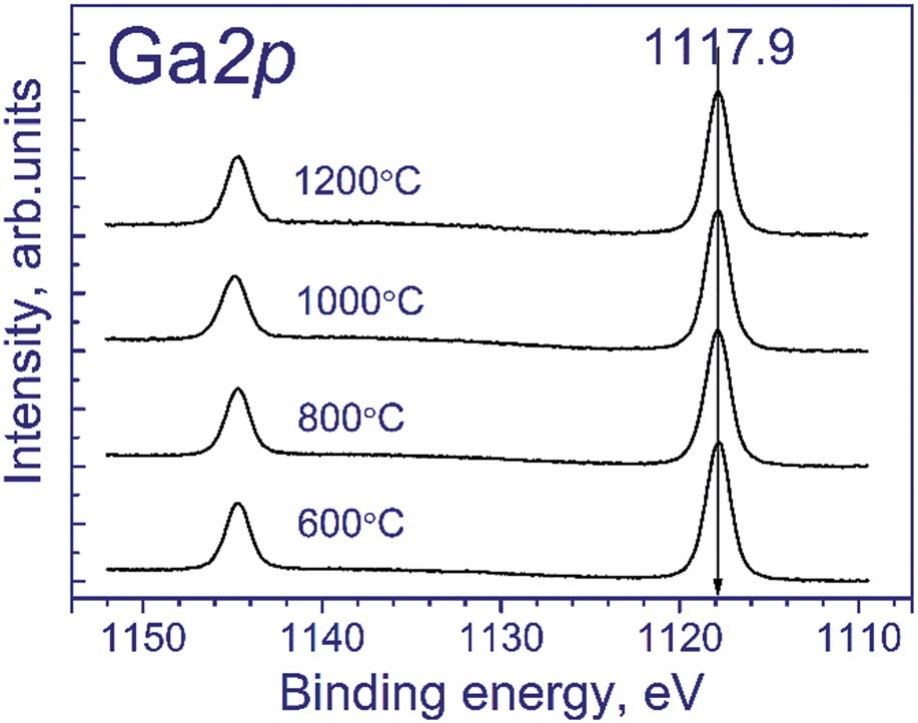
Ga 2p spectra of the studied samples.

Fig. 1 shows the Ga 2p spectra of the tested samples. The Ga 2p spectrum is represented by the Ga 2p_3/2_–Ga 2p_1/2_ spin-orbital doublet, and the integral intensity ratio of its lines is 2 : 1. The Ga 2p_3/2_ spectra of the tested samples are described by a single symmetric peak with the binding energy in the region of 1117.9 eV. According to the literature,^21–23^ position of the peak corresponds to gallium in the Ga^3+^ state. The state of manganese is commonly identified using the Mn 2p_3/2_ binding energy: according to the literature,^24–37^ manganese in MnO, Mn_2_O_3_ and MnO_2_ oxides is characterized by the Mn 2p_3/2_ binding energy in the range of 640.4–641.7, 641.5–641.9, and 642.2–642.6 eV, respectively. As seen from the indicated data, Mn^2+^ and Mn^3+^ compounds have close binding energies of Mn 2p_3/2_, which complicate their identification.

To identify correctly the manganese state, it is necessary to take into account also the shape of Mn 2p spectrum, specifically, the intensity and relative position of shake-up satellites, the presence of which is determined by multielectron processes.^26,28,38^ To estimate parameters of the Mn 2p spectra deconvolution into individual components, we recorded the spectra of reference compounds Mn_3_O_4_, Mn_2_O_3_, and MnO_2_, which contain manganese in the Mn^2+^, Mn^3+^ and Mn^4+^ states, respectively.

**Fig. 2:**
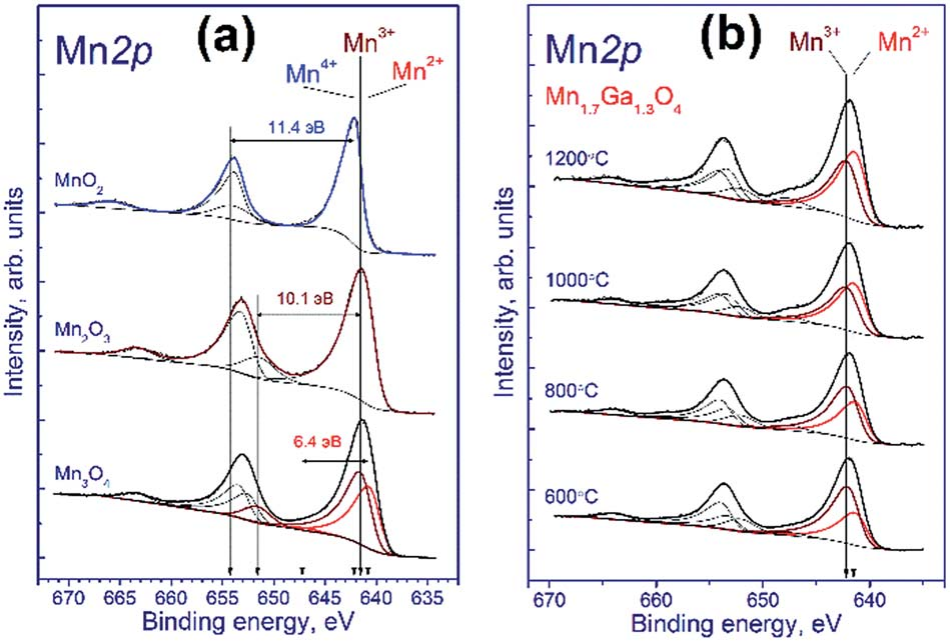
Mn 2p spectra of the studied samples. The spectra were normalized to the integral intensity of the corresponding Ga 2p spectra.

Fig. 2a shows the Mn 2p spectra of manganese oxides. It is known that the Mn 2p spectrum is represented by the Mn 2p_3/2_–Mn 2p_1/2_ spin-orbital doublet whose integral intensity ratio is 2 : 1 and spin-orbital splitting (the difference between binding energies of Mn 2p_1/2_ and Mn 2p_3/2_) is 11.8 eV. The Mn 2p spectrum of Mn_2_O_3_ comprises the Mn 2p_3/2_–Mn 2p_1/2_ doublet and the corresponding shake-up satellites; the binding energy of Mn 2p_3/2_ is 641.5 eV, and the difference in binding energy between the Mn 2p_3/2_ peak and the corresponding shake-up satellite is 10.1 eV. A similar pattern is observed for MnO_2_, the binding energy being equal to 642.2 eV, and the difference in binding energy between the Mn 2p_3/2_ peak and its shake-up satellite, 11.4 eV.

The Mn_3_O_4_ compound comprises both the Mn^2+^ and Mn^3+^ cations; therewith, the fraction of Mn^2+^ constitutes 33%, and that of Mn^3+^, 67%. The fitting of the Mn 2p spectrum revealed that in the synthesized Mn_3_O_4_ compound the fraction of Mn^2+^ constitutes 40%, and Mn^3+^, 60%, which is close to the stoichiometric composition. The Mn 2p_3/2_ binding energy of the Mn^2+^ state is 640.8 eV, and the difference in binding energy between the Mn 2p_3/2_ peak and the corresponding shake-up satellite, 6.4 eV. As was noted above, fitting parameters obtained for the model manganese compounds were used to identify the states of manganese in the tested Mn–Ga systems.

Fig. 2b shows the Mn 2p spectra of mixed Mn–Ga oxides treated in argon at different temperatures. The Mn 2p spectra are described by two Mn 2p_3/2_–Mn 2p_1/2_ doublets assigned to manganese in the Mn^2+^ and Mn^3+^ states, and by the corresponding shake-up satellites located at a distance of 6.4 and 10.1 eV from the main peaks. It should be noted that the Mn 2p spectra of the tested samples have no peaks located at a distance of 11.4 eV from the main peak, which is typical of the shake-up satellite of manganese in the Mn^4+^ state. In other words, manganese in the systems under consideration is in two states, Mn^2+^ and Mn^3+^. The Mn 2p_3/2_ peak assigned to manganese in the Mn^2+^ state lays at 641.5–641.6 eV, and that of manganese in the Mn^3+^ state, at 642.2–642.3 eV. Therewith, the indicated binding energies are higher than those observed for model manganese compounds Mn_3_O_4_ and Mn_2_O_3_ (640.8 eV for Mn^2+^ and 641.5 eV for Mn^3+^). A likely cause of the revealed differences is that manganese resides in the structure of mixed oxide whose local environment differs from the environment in simple oxide, and this produces the observed increase in the Mn 2p_3/2_ binding energy.

The relative concentrations (atomic ratios) of elements in the subsurface layer of the samples estimated by XPS and the Mn 2p_3/2_ binding energies are listed in Table 2. For the samples synthesized at 600–1000 °C, the [Mn]/[Ga] atomic ratio was 1.36–1.39, which is close to the stoichiometric composition 1.31, whereas for the sample treated at 1200 °C, a much higher atomic ratio, 1.87, was observed. Hence, an increase in the calcination temperature is accompanied by surface enrichment with manganese cations. For the low-temperature oxides treated at 600–800 °C, 67–60% of manganese cations in the 3+ state are observed, while for the high-temperature oxides this value is somewhat lower, ∼50%. For a compound with the composition Mn_1.7_Ga_1.3_O_4_, according to electroneutrality balance, Mn^3+^ should constitute 41%, and Mn^2+^ 59%; however, all the tested samples have a greater Mn^3+^ amount. Such behavior can be attributed to the oxidation on the manganese surface; but the presence of excess oxygen in the low-temperature oxides, as revealed by TG and XRD, should be taken into account as well.

**Table 2:** Atomic ratios of elements in subsurface layer of samples. The Mn 2p_3/2_ binding energies. The binding energy scale was calibrated using the Ga 2p_3/2_ peak at 1117.9 eV

Sample	[Mn]/[Ga]			Mn 2p_3/2_			
	Total	[Mn^2+^]/[Ga]	[Mn^3+^]/[Ga]	Mn^2+^	Mn^3+^	2+, %	3+, %
600 °C	1.37	0.45	0.91	641.6	642.2	33	67
800 °C	1.36	0.55	0.82	641.5	642.2	40	60
1000 °C	1.39	0.69	0.70	641.6	642.3	50	50
1200 °C	1.87	0.97	0.91	641.5	642.3	52	48

#### Catalytic activity in CO oxidation

**Fig. 3:**
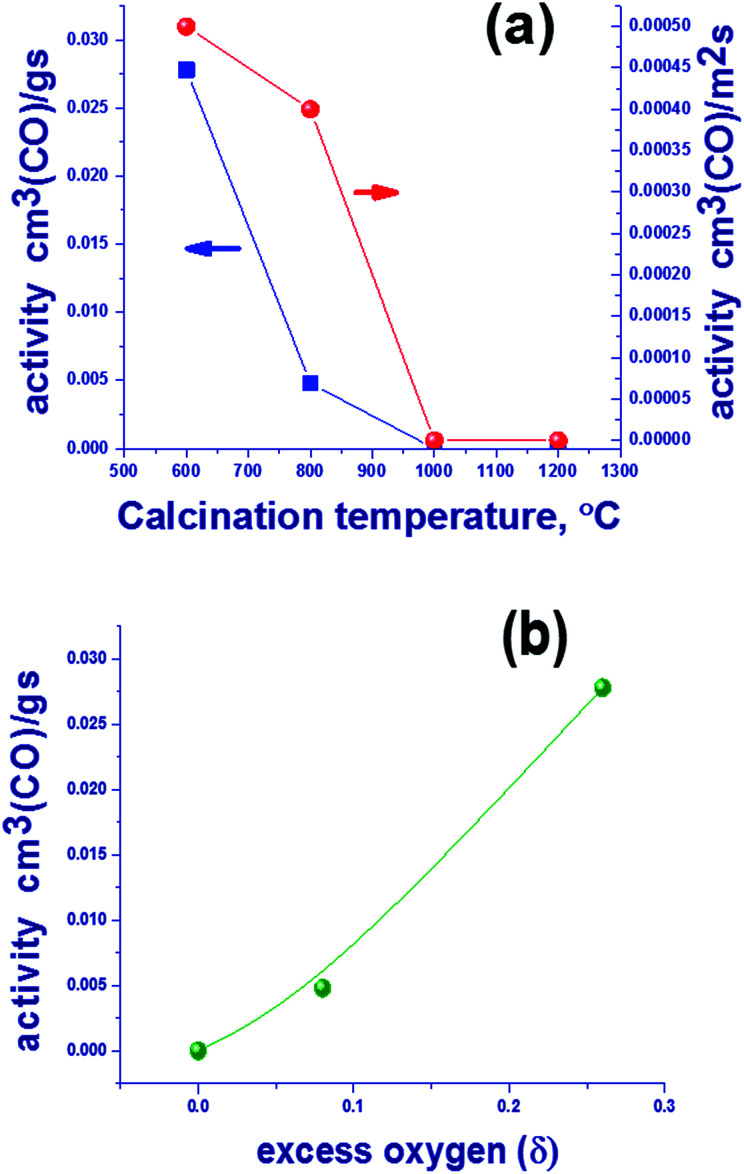
The catalytic activity of Mn–Ga oxides per gram of catalyst and per unit surface area in the oxidation of CO at 275 °C for oxides prepared at different temperatures (a), and the dependence of catalytic activity of the Mn–Ga oxides per gram of catalyst and quantity of excess oxygen according to TG data (b).

Fig. 3a shows the catalytic activity of Mn–Ga oxides per gram of catalyst and per unit surface area in the oxidation of CO as a function of calcination temperature. One can see that the catalytic activity per gram decreases to zero with increasing the temperature of oxide synthesis. The activity in the oxidation of CO referred to unit surface area also decreases when the calcination temperature is increased. It should be noted that non-zero activity in the oxidation of CO is observed for the samples containing excess oxygen. The content of excess oxygen correlates with the ratio of Mn^2+^/Mn^3+^ on the surface (Table 2). In addition, there is a correlation between the catalytic activity and the amount of excess oxygen *δ* in Mn_1.7_Ga_1.3_O_4+*δ*_ derived from TG data (Fig. 3b). Thus, characterization of the synthesized samples by XRD, TG, and XPS showed that the low-temperature (600–800 °C) oxides differ from the high-temperature (1000–1200 °C) ones by the presence of excess – nonstoichiometric oxygen, which is active in the catalytic oxidation of CO.

Since the spinel structure has the closest oxygen packing, the presence of additional oxygen should indicate the occurrence of defects – cationic vacancies. This suggests that an increase in the synthesis temperature results in a loss of excess oxygen, which decreases the number of cationic vacancies. Oxygen losses may be related not only to the oxidized surface (according to XPS) but also to the bulk structure, because an increase in the lattice parameters is observed (Table 1). Further studies are focused on the reduction of Mn–Ga and are aimed to reveal changes in the bulk and on the surface of the system during a loss of excess oxygen.

### Investigation of the hydrogen reduction of Mn_1.7_Ga_1.3_O_4_ spinel

#### TPR-H_2_

**Fig. 4:**
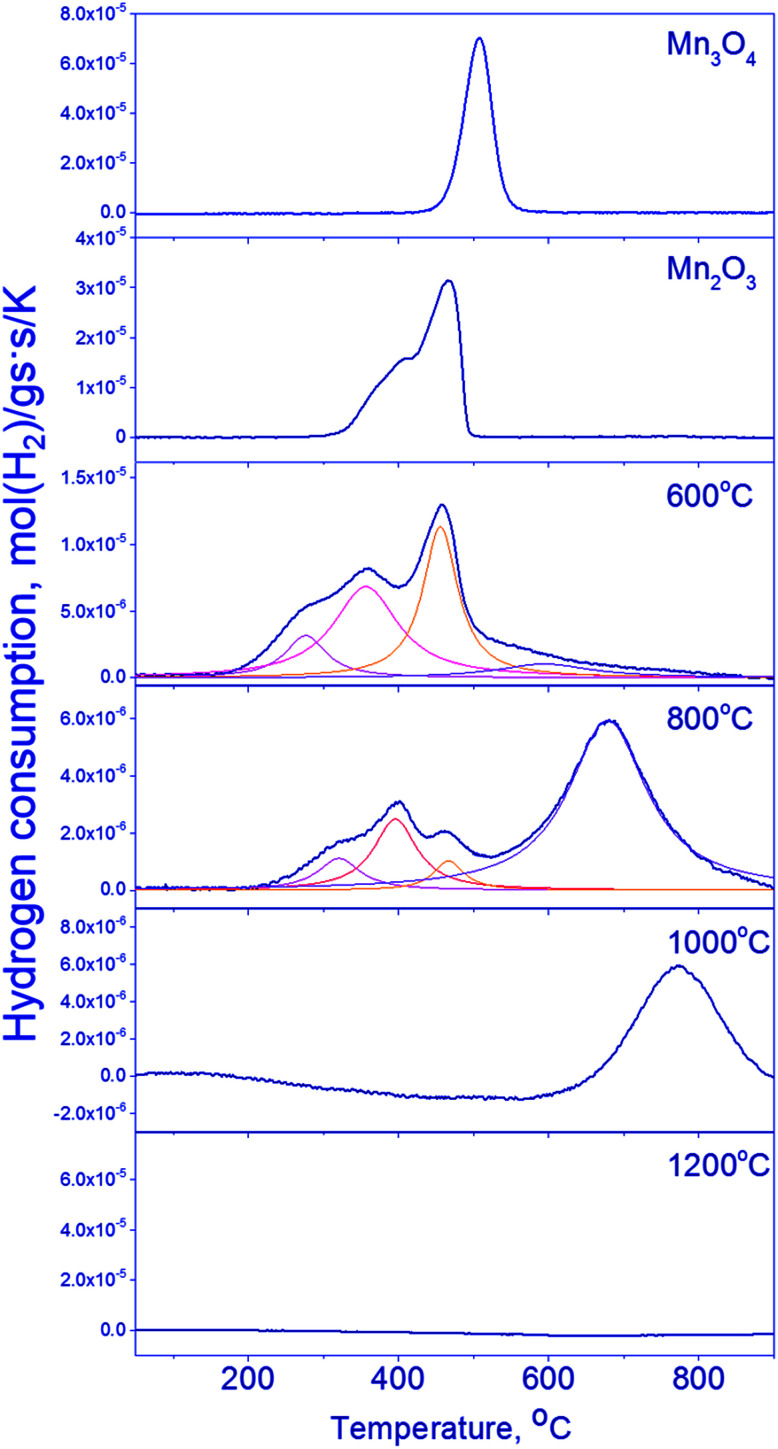
TPR profiles of Mn_1.7_Ga_1.3_O_4_ 600–1200 °C and simple oxides (Mn_2_O_3_ and Mn_3_O_4_).

Fig. 4 shows TPR curves for the series of samples under consideration; for comparison purposes, the corresponding data for Mn_2_O_3_ and Mn_3_O_4_ are also shown. One can see that the reduction of Mn_3_O_4_ is characterized by one hydrogen absorption peak with the maximum at 510 °C (Mn_3_O_4_ → MnO), whereas Mn_2_O_3_ is characterized by two peaks at 390 and 410 °C, corresponding to the two-step reduction Mn_2_O_3_ → Mn_3_O_4_ → MnO.^39,40^

The reduction of mixed Mn–Ga oxides differs essentially from the reduction of simple oxides. Low-temperature peaks at 280–470 °C and high-temperature peaks at 600–770 °C are observed on the TPR curves. Hydrogen absorption in two temperature ranges is typical of the samples synthesized at 600–800 °C. Therewith, the Mn_1.7_Ga_1.3_O_4_-800 °C sample has a pronounced hydrogen absorption peak at 680 °C; absorption in the region of 500–700 °C without a distinct maximum is observed on the curve of Mn_1.7_Ga_1.3_O_4_-600 °C. The TPR profile of the oxide treated at 1000 °C has only the high-temperature peak at 770 °C. Hydrogen absorption is not observed in Mn_1.7_Ga_1.3_O_4_-1200 °C; probably, it is reduced at higher temperatures (>900 °C).

For Mn_1.7_Ga_1.3_O_4_ compounds calcined at 600 and 800 °C, it can be supposed that TPR maxima in the low-temperature region correspond to the removal of weakly bound oxygen. Similar low-temperature TPR peaks were observed upon reduction of MnFe_2_O_4_, which was synthesized by the sol–gel method with subsequent calcination at 400–600 °C in air. The TPR curve had three peaks, which were assigned to the following reduction sequence: MnFe_2_O_4_ → MnFe_2_O_4−*δ*_ → MnO–FeO-solid solution → α-Fe.^41^

The higher is the calcination temperature, the stronger is the shift of hydrogen absorption peaks toward high temperatures, which produces a decrease in the total amount of absorbed hydrogen (Table 3).

**Table 3:** TPR data for Mn_1.7_Ga_1.3_O_4_ 600–1200 °C

Sample	*T*_max_	Amount of absorbed hydrogen, mmol g^−1^	Total hydrogen absorption, mmol g^−1^
600 °C	280	0.33	3.45
	355	1.09	
	455	1.04	
	600	0.99	
800 °C	320	0.15	2.07
	400	0.29	
	470	0.09	
	680	1.57	
1000 °C	770	0.93	0.93

The high-temperature TPR maximum at 600–800 °C obviously corresponds to the reduction of manganese cations to the Mn^2+^ state. Whether the reduction proceeds in the bulk of spinel or is accompanied by the release of MnO as a single phase – this question will be elucidated by means of *in situ* XRD and XPS.

#### *In situ* XRD

**Fig. 5:**
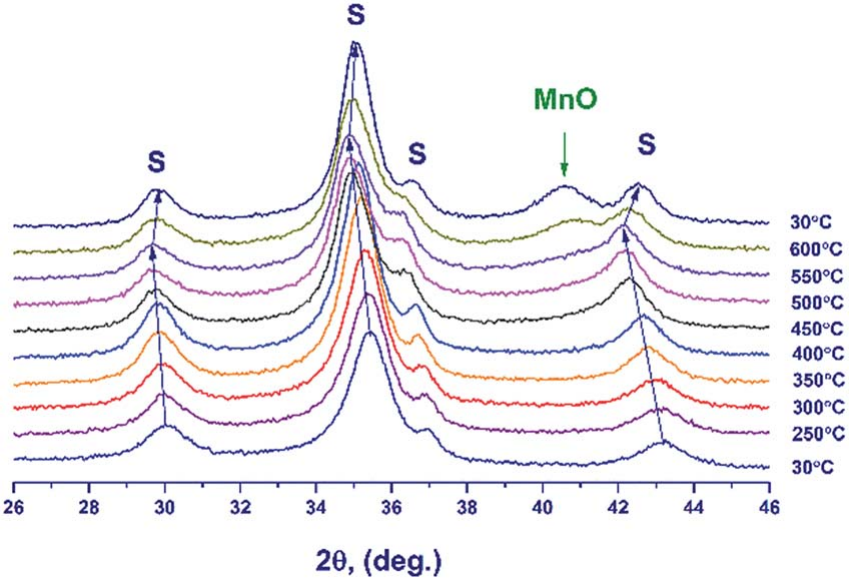
*In situ* XRD patterns recorded during the hydrogen reduction of Mn–Ga oxide synthesized at 600 °C in the temperature range from 30 to 600 °C. Reflections from Mn_1.7_Ga_1.3_O_4_ spinel are denoted by symbol S.

To reveal structural transformations accompanying the reduction of Mn–Ga mixed oxides, *in situ* XRD experiments were performed with heating of the sample synthesized at 600 °C in hydrogen (Fig. 5). As the temperature is raised, spinel peaks are shifted toward smaller angles; at 500–550 °C a shoulder appears near the spinel reflection in the region of 2*θ* = 41°; and at a further increase in temperature, this shoulder transforms into the peak at 2*θ* = 40.5° corresponding to the 200 reflection of MnO. During the reduction, spinel particles increased in size from 85 to 120 Å.

**Fig. 6:**
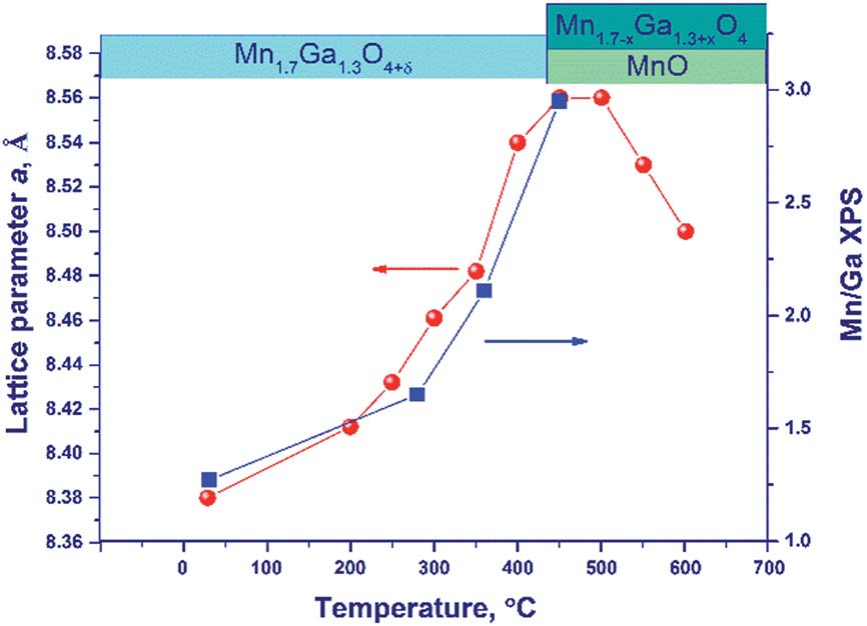
The lattice parameter of spinel (circles) and the [Mn]/[Ga] atomic ratio on the oxide surface (squares) *versus* temperature upon hydrogen reduction of the Mn–Ga oxide synthesized at 600 °C. The dynamics of phase transformations is illustrated by the diagram in the upper part of the figure.

Fig. 6 illustrates the temperature dependence of the spinel lattice parameters upon reduction. The lattice parameter increases from 8.413 to 8.560 Å in the range from 30 to 450 °C and then drops to 8.501 Å. The decrease in the lattice parameter is accompanied by the formation of the MnO phase.

For comparison purposes, Fig. 6 shows also the [Mn]/[Ga] atomic ratio on the surface as revealed by XPS, which will be discussed below.

An additional experiment was carried out to exclude the effect of thermal expansion: after heating in a hydrogen flow to 450 °C (until the MnO phase is formed) and to 600 °C, the lattice parameter was measured at room temperature.

In the temperature range of 25–450 °C, the unit cell parameter of spinel increases from 8.413(4) to 8.492(2) Å. The observed increase in the lattice parameter cannot be related to segregation of Mn cations on the surface (the XPS data are presented below) because an increase in the relative content of Ga^3+^ in the spinel bulk should decrease the lattice parameter. Thus, the observed change in the lattice parameter correlates with the loss of excess oxygen (the low-temperature TPR peak, Fig. 4). Nevertheless, noteworthy is the fact that the lattice parameter of spinel at 450 °C (8.492(2) Å) exceeds the parameters of the high-temperature oxides under consideration, 8.481(1)–8.478(1) Å (1000 and 1200 °C), although the indicated samples do not contain excess oxygen (according to the TG and TPR data). This may be related to rearrangements in the spinel structure and charge state of manganese cations.

At 600 °C the lattice parameter decreases to 8.454 Å, which is accompanied by the emergence of the MnO phase. The spinel parameter after reduction at 600 °C is close to the value indicated for Mn_1_Ga_2_O_4_, 8.451 Å [JCPDS no. 38-0181]. A decrease in the lattice parameter during the reduction at 500–600 °C is associated with the release of manganese cations from the solid solution to the MnO phase and with the increase in the relative Ga content in spinel. Accordingly, it can be expected that at 600 °C manganese cations are completely reduced to Mn^2+^ with the formation of MnGa_2_O_4_ and MnO phases.

Thus, the reduction is accompanied by changes in the lattice parameter, which testify to structural rearrangements caused by the removal of excess oxygen and the release of manganese cations with the formation of MnO oxide.

#### Pseudo *in situ* XPS analysis

To compare the behavior on the surface and in the bulk of the particle upon reduction, pseudo *in situ* XPS measurements were performed. Two experiments were carried out with the low-temperature sample: (1) treatment with argon at 200 and 400 °C, and (2) hydrogen reduction at the temperatures corresponding to the TPR maxima at 280, 360, and 450 °C. Table 4 lists the atomic ratios of elements in subsurface layer of the tested samples of mixed Mn–Ga oxide. Table 5 shows the Mn 2p_3/2_, Ga 2p_3/2_, and O 1s binding energies as well as the content of Mn^2+^ and Mn^3+^ cations.

**Table 4:** Atomic ratios of elements in the subsurface layer of Mn–Ga oxide synthesized at 600 °C under different treatment conditions^a^

Treatment (temperature and medium)	[Mn]/[Ga]			[O*]/[Mn]	[O*]/[Ga]	[O*]/[Mn] + [Ga]
	Total	[Mn^2+^]/[Ga]	[Mn^3+^]/[Ga]			
**Treatment in Ar**						
Fresh	1.37	0.45	0.91	2.45	3.35	1.4
200 °C-Ar	1.48	0.73	0.75	2.3	3.4	1.4
400 °C-Ar	1.54	0.96	0.58	1.9	2.9	1.2

**Treatment in H**_**2**_						
280 °C-H_2_	1.81	1.04	0.77	2.31	4.17	1.5
360 °C-H_2_	2.33	1.00	1.33	1.39	3.23	0.97
450 °C-H_2_	3.26	1.22	2.04	1.24	4.03	0.95

a [O*] – oxygen in the structure of mixed oxide.

**Table 5:** Mn 2p_3/2_ and Ga 2p_3/2_ binding energies (eV). The binding energy scale was calibrated against the Ga 2p_3/2_ peak at 1117.9 eV

Treatment (temperature and medium)	Mn 2p_3/2_				Ga 2p_3/2_	O 1s
	Mn^2+^	Mn^3+^	Mn^2+^, %	Mn^3+^, %		
**Treatment in Ar**						
Fresh	641.6	642.2	33	67	1117.9	530.6
200 °C-Ar	641.0	642.2	49	51	1117.9	530.6
400 °C-Ar	640.9	642.3	62	38	1117.9	530.6

**Treatment in H**_**2**_						
280 °C-H_2_	641.1	642.2	58	42	1117.9	530.6
360 °C-H_2_	641.1	641.8	43	57	1117.9	530.6
450 °C-H_2_	641.1	641.8	37	63	1117.9	530.3

As for the initial samples, the Ga 2p spectra of Mn–Ga oxide (600 °C) recorded after treatment in argon and reduction in hydrogen are described by one symmetric peak at 1117.9 eV (spectra not shown), which corresponds to Ga^3+^.^19–21^ This peak was used for calibration of the binding energy scale.

**Fig. 7:**
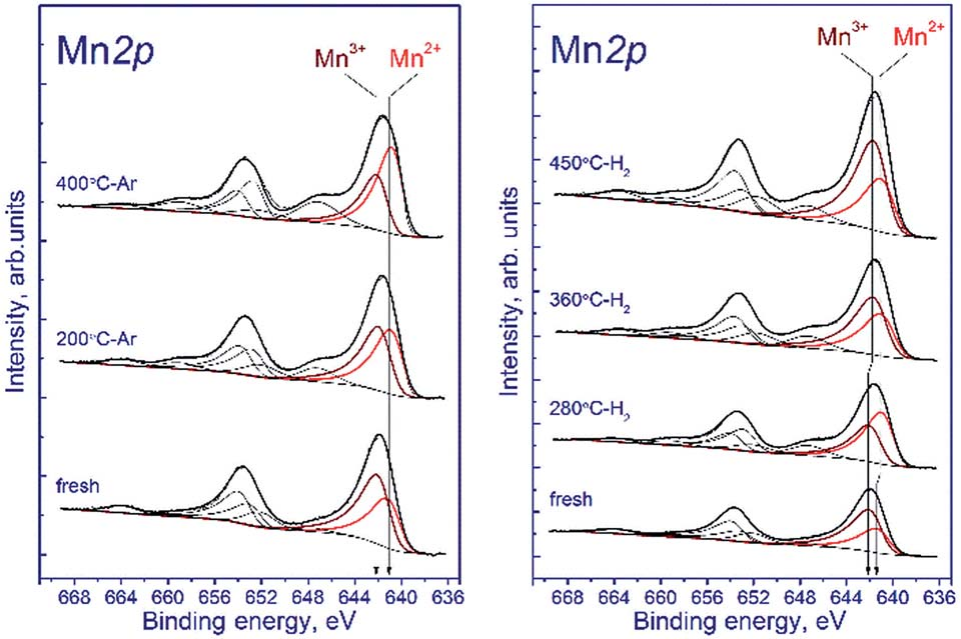
Mn 2p spectra of the studied samples. The spectra were normalized to the integral intensity of the corresponding Ga 2p spectra.

Fig. 7a shows the Mn 2p spectra of the tested sample that were recorded after the treatment in argon. According to deconvolution of the Mn 2p spectra of the sample into individual components, manganese is in two states, Mn^2+^ and Mn^3+^. As the treatment temperature is raised, the Mn 2p_3/2_ peak attributed to manganese in the Mn^2+^ state shifts from 641.5 to 640.9 eV. A possible reason is the formation of the manganese-containing particles on the sample surface in which the chemical environment of manganese is close to that in MnO. The Mn 2p_3/2_ peak assigned to manganese in the Mn^3+^ state lays at 642.2–642.3 eV indicating that Mn^3+^ cations reside in the structure of a mixed Mn–Ga oxide.

Relative concentrations (atomic ratios) of elements in the subsurface layer, which were estimated by XPS, are listed in Table 5. The treatment in argon decreases the oxygen content: the [O]/[Mn] atomic ratio droppes from 2.45 to 1.9. Simultaneously, the [Mn]/[Ga] atomic ratio increases from 1.3 to 1.5, which indicates that manganese is segregated as MnO on the surface of mixed oxide. This is evidenced also by an increase in the fraction of Mn^2+^ from 33% to 62%. On the surface, the [Mn^2+^]/[Ga] ratio increases from 0.48 to 0.96, while [Mn^3+^]/[Ga] decreases from 0.91 to 0.58 (Table 4). Surface enrichment with manganese is also observed for the initial sample calcined at 1200 °C, in comparison with other samples of this series (Table 2). The [O]/[Mn + Ga] ratio decreases from 1.4 to 1.2, which agrees with the weight losses and the removal of excess oxygen revealed by TGA.

Fig. 7b shows the Mn 2p spectra of the tested sample that were recorded after its reduction in hydrogen. The shape of the Mn 2p spectra indicates that manganese is in two states, Mn^2+^ and Mn^3+^. The presence of manganese in the Mn^4+^ state is not observed. Similar to the treatment in argon, the treatment in hydrogen at 280 °C leads to the shift of the Mn 2p_3/2_ peak attributed to Mn^2+^ from 641.6 to 641.0 eV; this fact testifies to the formation of the manganese-containing particles on the catalyst surface in which the chemical environment of manganese is close to that in MnO. The Mn 2p_3/2_ peak assigned to Mn^3+^ also shifts from 642.3 to 641.8 eV; however, the observed effect takes place at 360 °C, which may indicate the formation of individual particles containing manganese in the Mn^3+^ state (probably Mn_2_O_3_). In both cases, the decrease in the Mn 2p_3/2_ binding energy is accompanied by an abrupt increase in the [Mn^2+^]/[Ga] and [Mn^3+^]/[Ga] atomic ratios.

The surface content of Mn^2+^ increases from 33% to 58% upon reduction in hydrogen at 280 °C and then decreases to 43% (360 °C) and 37% (450 °C) when the temperature is raised. The decrease in the relative content of Mn^2+^ on the surface during the hydrogen reduction seems unusual, because the process occurs in a reducing atmosphere and should be accompanied by a decrease in the oxidation state of manganese cations, as in the case of treatment in argon. However, the reduction is accompanied by segregation of manganese cations on the surface; therewith, the [Mn^2+^]/[Ga] atomic ratio gradually increases from 0.45 to 1.22 upon heating in hydrogen from 25 to 450 °C. The [Mn^3+^]/[Ga] atomic ratio changes abruptly during the reduction: it decreases from 0.92 to 0.77 upon heating from 25 to 280 °C, then sharply increases to 1.3 at 360 °C, and reaches 2.0 at 450 °C.

Upon reduction of the sample in hydrogen at different temperatures, the [Mn]/[Ga] atomic ratios increase from 1.4 to 3.3, and the [Mn^2+^]/[Ga] and [Mn^3+^]/[Ga] atomic ratios also increase, indicating the segregation of manganese on the surface. This fact is also supported by a decrease in the [O]/[Mn + Ga] atomic ratio (Table 4). Fig. 6 sums up data on the [Mn]/[Ga] atomic ratio in the subsurface layer as compared to the bulk characteristic – the lattice parameter of spinel in dependence on temperature; a correlation between the XRD and XPS data is seen.

Hence, the XPS data demonstrate that the treatment in argon or in hydrogen produces changes in the surface composition: manganese cations Mn^2+^ segregate, and the [O]/[Mn + Ga] atomic ratio decreases. It means that the removal of excess oxygen is accompanied not only by structural rearrangement but also by surface modification. At the same time, changes in the Mn^2+^/Mn^3+^ surface ratio that occur in the hydrogen atmosphere seem unusual: an increase in the concentration of Mn^3+^ and a decrease in Mn^2+^; however, they allow proposing a scheme of the initial step of reduction.

#### The mechanism of reduction

Changes in the Mn^2+^ and Mn^3+^ content, the [Mn]/[Ga] atomic ratio on the sample surface, and bulk characteristics of the oxide revealed by XRD give grounds to suggest the mechanism of reduction in the first (initial) step. It should be reminded that the evolution of the surface composition at temperatures up to 450 °C is not accompanied by changes in the phase composition, and only a loss of excess oxygen is observed. Upon the treatment in hydrogen, oxygen is removed from the surface, and the surface Mn^3+^ cations are reduced to Mn^2+^. In spite of the observed segregation of manganese cations, the produced surface Mn^2+^ cations ‘diffuse’ into the bulk (most likely the hole is displaced), thus decreasing the relative content of Mn^2+^ on the surface upon reduction (Table 5). This process is implemented when the ‘diffusion’ rate of Mn^2+^ cations substantially exceeds the reduction rate. The emergence of Mn^2+^ cations into the bulk may testify to the formation of MnO clusters in the initial compound, which will pass to the MnO phase upon a further increase of temperature. The appearance of MnO clusters in the initial spinel structure indicates the substitution of the Mn^2+^ cations for Mn^3+^ and Ga^3+^ cations in octahedral positions or the filling of internode spaces. The ionic radius of Mn^2+^ in the octahedral environment, which is equal to 0.83 Å, is greater than the corresponding values for Mn^3+^ (0.58 Å) and Ga^3+^ (0.62 Å); this increases of the lattice parameter, MnO phase is released from the structure of initial oxide, and the relative content of Mn in spinel decreases to MnGa_2_O_4_.

**Fig. 8:**
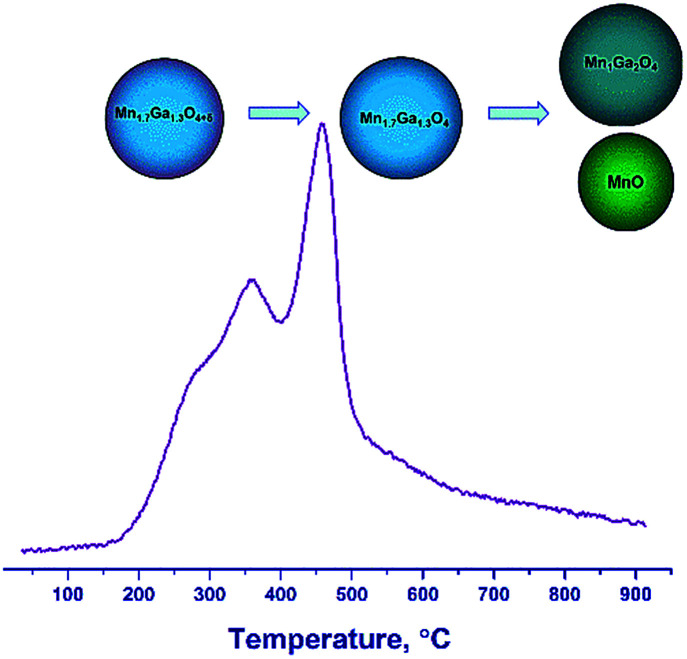
A scheme of transformations upon reduction of Mn_1.7_Ga_1.3_O_4+*δ*_ by hydrogen.

Therefore, the hydrogen reduction of Mn–Ga oxides with the spinel structure in the temperature range of 30–900 °C can be presented as follows. For the samples synthesized at 600–800 °C, a two-step reduction of the oxide is observed, which is indicated by changes in the lattice parameter and by the TPR data. According to TG and TPR, the initial samples contain a certain amount of excess oxygen, Mn_1.7_Ga_1.3_O_4+*δ*_, as compared to the stoichiometric content. In the first step, excess oxygen is removed, which is accompanied by an increase in the lattice parameter. The increase in the lattice parameter correlates with changes in the surface composition (the enrichment with manganese cations) revealed by XPS. In the second step, the Mn^3+^ cations are reduced to Mn^2+^, and manganese cations pass from the spinel structure to the MnO phase, thus changing the spinel composition from Mn_1.7_Ga_1.3_O_4_ to MnGa_2_O_4_ (Fig. 8).

For the high-temperature samples synthesized by calcination at 1000–1200 °C in argon, a single-step reduction of the oxide is observed. Initially, Mn cations are formally in the oxidation states Mn^2+^ (1.0) and Mn^3+^ (0.7) according to the formula Mn_1_^2+^Mn_0.7_^3+^Ga_1.3_^3+^O_4_; as a result of reduction, all Mn^3+^ cations turn into Mn^2+^. Probably, redistribution of Mn and Ga cations over tetrahedral and octahedral positions, respectively, with the formation of Mn_1_Ga_2_O_4_ spinel and the release of the MnO phase, becomes favorable.

## Conclusions

A series of Mn_1.7_Ga_1.3_O_4_ mixed oxides synthesized by coprecipitation with subsequent calcination in argon at 600–1200 °C has been studied. TG and TPR studies revealed the presence of excess oxygen in the samples synthesized at 600–800 °C. The loss of oxygen upon heating in argon (according to TG) and the presence of low-temperature peaks of hydrogen absorption (according to TPR) are observed for such oxides. An increase in the synthesis temperature to 1000–1200 °C results in the formation of mixed oxides not containing superstoichiometric oxygen. A correlation was found between the content of excess oxygen in the Mn–Ga oxides and their catalytic activity in the oxidation of CO.

The process of hydrogen reduction of the Mn–Ga oxides with the spinel structure has been studied. For the samples synthesized at 600–800 °C, a two-step reduction of the oxide is observed. According to TPR and *in situ* XRD studies, excess oxygen is removed in the first step. An increase in the lattice parameter correlates with changes in the surface composition revealed by XPS, which indicates that changes in the bulk of the oxide modify the sample surface. The removal of excess oxygen upon reduction (which shows up as an increase in the lattice parameter) is accompanied by surface enrichment with manganese cations. In the second step, Mn^3+^ cations are reduced to Mn^2+^ in the spinel structure with the release of the MnO phase.

## Conflicts of interest

There are no conflicts to declare.

## Supplementary Material
